# Evaluating the effects of multimodal EEG-fNIRS neurofeedback for motor imagery: An experimental platform and study protocol

**DOI:** 10.1371/journal.pone.0331177

**Published:** 2025-09-11

**Authors:** Camille O. Muller, Thomas Prampart, Elise Bannier, Isabelle Corouge, Pierre Maurel

**Affiliations:** 1 Univ Rennes, Inria, CNRS, Inserm, IRISA UMR, Empenn E.R.L. U., Rennes, France; 2 Inria, Univ. Rennes, CNRS, IRISA, Rennes, France; 3 CHU Rennes, Department of Radiology, Rennes, France; Air University, PAKISTAN

## Abstract

**Background:**

Neurofeedback (NF) enables the self-regulation of brain activity through real-time feedback extracted from brain measures. Recently, the combination of several neuroimaging methods to characterize brain activity has led to growing interest in NF. The integration of various portable recording techniques, such as electroencephalography (EEG) and functional near-infrared spectroscopy (fNIRS), respectively based on electrical and hemodynamic activity, could enhance the characterization of brain responses and subsequently improve NF performance. Such multimodal NF used with motor imagery (MI) could benefit post-stroke motor rehabilitation to stimulate neuroplasticity of the lesioned motor areas. Nevertheless, their concomitant use in NF to identify brain activity features during upper-limb MI-based NF has not been studied to our knowledge. The objective of this paper is to present our fully operational experimental platform and the study protocol we propose to assess the benefits of combining EEG and fNIRS for NF in the context of MI.

**Methods:**

We developed a custom experimental platform, including a cap integrating EEG and fNIRS sensors, along with software for real-time signal processing, NF score calculation and visual feedback presentation. To evaluate the effect of EEG-fNIRS NF in the context of MI, thirty right-handed participants will undergo three randomized NF conditions: EEG-only, fNIRS-only and EEG-fNIRS based NF. EEG electrodes and fNIRS channels will be positioned above the sensorimotor cortices. Participants will be presented with a visual representation of a ball along a one-dimensional gauge moving upwards according to their brain activity level as they perform a MI task of their left-hand. An NF score will be computed from the right primary motor cortex activity along the three different experimental conditions (EEG-only, fNIRS-only, EEG-fNIRS). The association between the NF score, the neuroimaging modality and the motor imagery strategy will then be analyzed.

**Discussion:**

The designed experimental platform allows combined EEG-fNIRS NF. The presented study protocol will be the first investigation of the effects of multimodal neurofeedback using EEG and fNIRS during upper-limb MI tasks. We hypothesize that presenting the participants with a visual NF representing a score based on both EEG and fNIRS signals will result in more specific task-related brain activity in the sensorimotor cortices. With potentially increased neuroplasticity, such a system could find applications in clinical contexts, particularly in motor rehabilitation, for example in post-stroke units.

## 1. Background

Neurofeedback (NF) is a neuroscientific method that aims to enhance brain efficiency. NF enables the self-regulation of brain activity through real-time feedback (e.g., visual, auditory or other sensory representations) extracted from brain measures [1]. Neurofeedback can lead to measurable and lasting changes in brain activity, reflecting functional plasticity [2]. This process is often conceptualized in terms of operant conditioning, where successful modulation of neural activity is positively reinforced [3]. This process may lead to improvements in behavior, physical health, cognition and emotional functioning [3–6]. NF has been explored for a variety of clinical [7–10] and non-clinical applications [3–6,11,12]. However, its effective integration into clinical practice is hindered by ongoing debates over its efficacy, likely due to issues such as poor study design, a lack of standardized guidelines and limited understanding of its underlying mechanisms [12,13]. One reason for the contested efficacy of current approaches might be the inherent limitations of single imaging modalities [14,15].

As NF focuses on the activity of the central nervous system, a key challenge in its application is to determine which neuroimaging method and strategy to use (e.g., NF metaphor, mental task, design). Neurofeedback studies often report that approximately 30% of participants face difficulties in self-regulating their brain activity [16]. These challenges may arise from suboptimal feature selection, design flaws in feedbacks, tasks and instructions [17,18], individual anatomical and physiological differences that reduce responsiveness to specific modalities [19]. Each neuroimaging modality captures specific biophysical aspects of brain activity but is subject to different technical and physiological limitations [14]. The field of NF originates from electroencephalography (EEG), which measures rapid changes in electrical potentials across the scalp and is valued for its portability, non-invasiveness and high temporal resolution [20]. On the other hand, over recent decades, researchers have explored neurovascular coupling through functional magnetic resonance imaging (fMRI; [21]) and more recently, through functional near-infrared spectroscopy (fNIRS) which tracks changes in oxygenated (HbO_2_) and deoxygenated (HbR) hemoglobin in cortical regions [22,23]. Both fMRI and fNIRS methods offer higher spatial resolution but lower temporal resolution than EEG. Compared to fMRI, fNIRS is a cost-effective, portable and adaptable tool for hemodynamic-based real-time applications in an ecological context. Nevertheless, fNIRS also presents a lower spatial resolution and limited depth but offers a higher temporal resolution. While unimodal techniques provide valuable insights, their limitations can be mitigated by combining complementary modalities which is really promising in the field of brain computer interface (BCI) to decode brain activity [24,25].

Recently, there has been growing interest in integrating multiple brain activity measurement technologies. The integration of these technologies offers a novel approach that leverages the strengths of each while addressing their individual limitations [26]. This concept involves combining various recording techniques to enhance the performance of NF, with studies demonstrating the advantages of multimodal acquisition systems compared to unimodal ones [27,28]. The most prevalent multimodal system involves the concurrent use of fMRI and EEG. This combination capitalizes on EEG high temporal resolution and fMRI superior spatial resolution, providing a complementary approach to neurofeedback [27,29–32]. It has also been tested in post-stroke clinical contexts [33,34]. However, this approach has significant drawbacks related to fMRI inherent limitations, such as high operating and acquisition costs, the contraindications related to the use of fMRI and the need for almost total immobilization of the patient in a lying position [35].

Another multimodal system under development is fNIRS-EEG, a mobile setup that facilitates use in ecological contexts, such as clinical environments [36]. EEG and fNIRS share mutual information by picturing the cortical brain activity, yet also exhibit important distinct features with for example the recording of electrical activity and hemodynamic variations. For example, the study by Fazli et al. [15] demonstrated that EEG and fNIRS complement each other in terms of information content with information gained from various sources, making them a viable multimodal imaging technique suitable for BCI. Regarding the association of fNIRS and EEG in NF, Zich et al [37] proposed a multimodal neuroimaging framework that focuses on EEG-derived event-related desynchronization (ERD, i.e., decrease in neural oscillatory power activities within EEG signals during movement execution, [38]) and fNIRS-based measures of HbO_2_ and HbR. They identified significant modulation correlations between ERD and hemodynamic measures, despite the absence of significant amplitude correlations. However, in their study, the authors used the fNIRS signal for offline analysis presenting EEG unimodal NF in real-time only [37]. A review exploring fNIRS-EEG-based BCIs suggests that combining peak and mean fNIRS signals with the highest band powers of EEG signals is promising for multimodal BCIs to improve the system accuracy [39]. In the context of eating disorders, another study demonstrated that EEG- and fNIRS-based NF produced similar outcomes, although the authors did not integrate both signals into a unified NF approach [40]. Finally, simultaneous measurement of fNIRS and EEG is easy to combine and use in neuroimaging studies for other applications (for reviews see [40,41]). Therefore, it could be highly relevant to apply this combination to NF and develop methodologies for real-time analysis of both signals.

Although multimodal EEG-fNIRS is considered to combine the advantages of EEG and fNIRS in a way that compensates for the limitations of each modality, its potential to record cerebral activity features has not been comprehensively investigated [26]. In motor rehabilitation, motor imagery (MI) – the mental representation of an action without its actual execution [42] – is commonly incorporated into NF protocols to target brain motor areas, making it a suitable task for anticipating later clinical application [9]. To our knowledge, no study has explored the effect of multimodal EEG-fNIRS-based NF in the context of MI. Thus, the aim of this paper is to evaluate the efficiency of EEG-fNIRS-based NF for MI in comparison to unimodal EEG-based and fNIRS-based NF. To reach this objective, we developed and implemented an experimental platform for multimodal EEG-fNIRS NF in the context of upper limb (UL) MI. Implementing such multimodal systems presents significant challenges, including the need for seamless coordination and synchronization of two inherently distinct systems, while maintaining real-time performance and ensuring that they do not interfere with each other. We then designed a randomized controlled study with healthy volunteers to assess the potential of multimodal EEG-fNIRS NF. We hypothesize that presenting participants with visual NF based on a combined score from both EEG and fNIRS signals will result in higher sensorimotor brain activity reflected in both EEG and fNIRS signals. As exploratory outcomes, we will investigate the participants’ feeling of NF control, the relationship between the motor imagery vividness (i.e., ability to build image and kinesthetic sensation of a movement) and the NF score. We will start by presenting the designed and already operational experimental platform and will then present the study protocol proposed to evaluate the effects of EEG-fNIRS based NF.

## 2. Methods

### 2.1. Experimental platform

In order to evaluate the effect of multimodal EEG-fNIRS NF for MI of the left upper limb, we designed and implemented an experimental platform. The experimental platform was implemented for block design protocols and requires a step of calibration for parameterization of the score calculation. This platform contains a homemade cap-layout combining EEG and fNIRS sensors specifically designed for our NF target and an application software to receive and process signals in real-time, calculate an NF score and present the user with a visual NF. The source code is accessible from a git repository (https://gitlab.inria.fr/empenn-public/neurofeedback/fNIRS-EEG-NFB) and archived on Software Heritage (swh:1:dir:6433906c87451098cd3e324215715ba552396399).

#### 2.1.1. *Cap-layout.*

**Fig 1:**
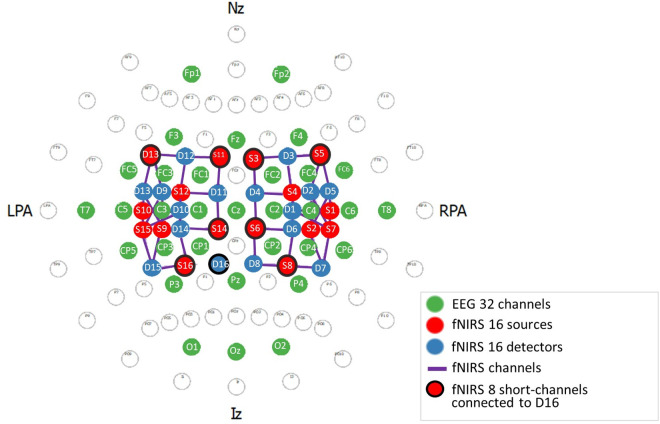
Set-up designed for our NF-platform. Brain cap layout on the 10−10 international system. Nz (Nasion), Iz (Inion), LPA (left pre-auricular), RPA (right pre-auricular). EEG channels are in green, fNIRS detectors in blue, fNIRS sources in red and fNIRS channels in purple. The short channels are connected to detector 16 and their sources are shown in red surrounded by a black circle.

To simultaneously record EEG and fNIRS data, we designed a cap-layout using a 32-channel EEG system (ActiCHamp, Brain Products GmbH, Gilching, Germany) and a continuous wave NIRS system with 16 detectors, 16 LED sources (λ1|2 = 760|850 nm) and 8 short channels (NIRScout XP, NIRx, Berlin, Germany). All sensors are installed on an EasyCap (Easy-cap, CNX-128 Cap, Brain Products GmbH, Gilching, Germany). Based on the 10−10 international system, we positioned 19 of the 32 EEG channels above the sensorimotor cortices (FC5, FC3, FC1, FC2, FC4, FC6, C5, C3, C1, C2, C4, C6, CZ, CP5, CP3, CP1, CP2, CP4, CP6). Additionally, three electrodes are placed above the occipital cortices (OZ, O1, O2), four above the frontal areas (Fp1, Fp2, F3, F4, Fz), two above the temporal regions (T7, T8) and three above the parietal region (Pz, P3, P4). For the fNIRS channels, we positioned the optodes over both sensorimotor cortices, with 22 long channels (source-detector distance = 3 cm) and 4 short channels (source-detector distance = 0.8 cm) over the left sensorimotor area (SM), and 24 long and 4 short channels over the right SM. The difference in the number of channels is due to the use of detector 16 (D16) to connect the 8 short channels. Fig 1 shows the complete layout of all EEG and fNIRS sensors on the cap.

#### 2.1.2. *NF features real-time preprocessing.*

**EEG.** In our platform, the EEG feature for NF is defined as the ERD in the alpha band (8–13 Hz) of the EEG data. The raw EEG signal undergoes several preprocessing steps. First, Fz is set as the reference electrode. Next, only the electrodes that superimpose the brain area of interest are kept (in our subsequent study, we will consider C4, FC2, FC6, CP2 and CP6). Then, a spatial filter (Laplacian, 43) is applied to enhance the signal from the electrode representing the NF target, here C4 (i.e., left hand representation in the primary motor cortex, [43,44]). The filter coefficients are: C4 (4), FC2 (−1), FC6 (−1), CP2 (−1), and CP6 (−1). To calculate a weighted difference between the signal at the central channel and the signals from its immediate surrounding neighbors, a positive coefficient (+4) is assigned to the central channel (C4), and negative coefficients (−1) are assigned to its surrounding neighboring channels (FC2, FC6, CP2, CP6). A band-pass filter (order 4, cutoff frequencies 8–13 Hz) is then applied to the resulting signal in order to keep only the alpha-band that is used to calculate the NF score.

**fNIRS.** The fNIRS feature is based on the oxyhemoglobin change in concentration (ΔHbO_2_) computed from the two channels located above the target EEG channel (i.e., D1-S1; D2-S2 channels in the subsequent protocol study, see **Fig 1**). First, the raw intensity data are converted in real-time to optical density using the last 120 s of the signal [45]. Next, to remove extracortical components from the signal, a correction is performed using the short-channel signals by doing a systemic correction regression based on the nearest short channel [46–48]. We choose to apply the short-channel correction on the optical density value as in previous fNIRS-BCI studies [49]. Then, the corrected optical density signal is converted to variations in HbO2 and HbR concentrations using the modified Beer-Lambert law ([50]; ppf = 6.0). A band-pass filter (order 4, 0.01–0.09 Hz) is finally applied to remove potential confounding signals such as Mayer waves, heartbeat and breathing [49,51,52].

#### 2.1.3. *Neurofeedback score.*

**Fig 2:**
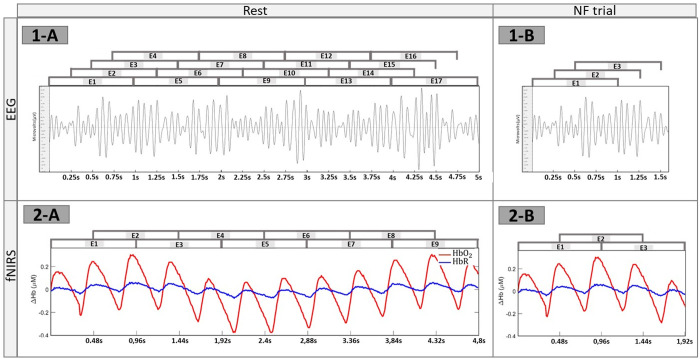
Epoching of the EEG and fNIRS signals. 1-A represents the epochs averaged for the rest period and 1-B for the task for the EEG signal. 2-A represents the epochs averaged for the rest period and 2-B for the NF task for the fNIRS signal.

**EEG-NF score.** A time-based epoching is applied to the EEG preprocessed signal to generate 1 s epochs occurring every 0.25 s. Then, the power of the EEG signal is computed. An EEG-NF score is calculated every 0.25 s based on 1.5 s periods (i.e., averaged over 3 epochs) using a 5 s rest periods (i.e., average of 17 epochs, see Fig 2). In order to prevent from temporal drift, the EEG-NF score is computed considering the last 5* seconds of the previous rest period as the baseline. The ERD ratio is then calculated:

$Ratio\;ERD=\frac{\left(pTask\;-pRest\right)}{pRest}*100$, where *pRest* represents the power averaged from the epochs from the last 5 s of the preceding rest period and *pTask* represents the power averaged from the last 1.5 s that capture the near-instantaneous power during the NF task [53]. A new ERD ratio is calculated every time a new epoch is received (i.e., every 0.25 s according to the time based epoching). During the calibration session, the 30th percentile of the ERD ratios is extracted from all the calibration blocks and set as the target level to reach 100% during the NF session. This choice was made based on previous NF protocols, in order to individually set the difficulty of the NF task (i.e., maximal value of the gauge). We chose to focus solely on the expected movement-related desynchronization, which is indicated by a negative ERD during the task [54]. This value is stored and used to compute the EEG-NF score: $NF_{EEG}=\;\frac{Ratio\;ERD\;(NF\;task)}{Ratio\;ERD\;(calibration)}*100$.

**Fig 3:**
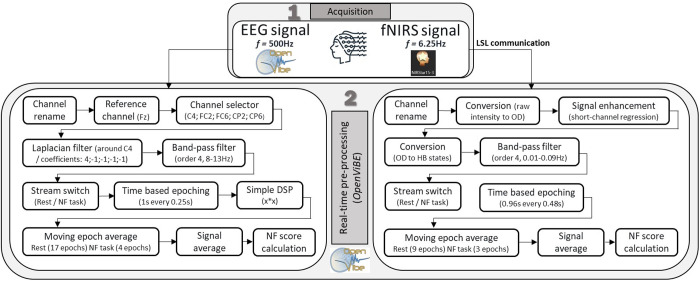
Implementation of EEG and fNIRS from acquisition and real-time preprocessing of EEG and fNIRS signals to the calculation of the NF score. 1. Acquisition is done through the OpenViBE acquisition server for the EEG, and for the fNIRS the raw signal is recorded via the NIRStar software and transferred to the openViBE acquisition software via LSL communication. 2. Real-time preprocessing steps are conducted in the OpenViBE designer software.

**fNIRS**-**NF score.** A time-based epoching is applied to generate epochs of 0.96 s every 0.48 s for the fNIRS (see Fig 3). The duration of the epochs and moving window of the fNIRS is based on the sampling frequency (i.e., 6.25 Hz), ensuring a constant and integer number of fNIRS samples across all epochs. A fNIRS NF score is calculated every 0.48 s based on 1.92 s periods (i.e., averaged of 3 epochs) using a 4.8 s rest periods (i.e., average of 9 epochs, see Fig 2). In order to prevent from temporal drift, the fNIRS-NF score is computed considering the last 4.8* seconds of the previous rest period as the baseline. The **diff*Δ*HbO**_*2*_ is then calculated as followed:

**diff*Δ*HBO**_*2*_* = ΔHBO*_*2(Task)*_ **-* Δ*HBO**_*2(Rest*)_*,* where *ΔHBO*_*2(Rest)*_ represents the average of the epochs from the last 4.8 s of the preceding rest period and *ΔHbO*_*2(Task)*_ represents the average over the last 1.92 s reflecting the near-instantaneous relative variation in oxyhemoglobin concentration during the NF [49]. A new ΔHbO_2_ value will be calculated every 0.48 s. To follow the EEG targets, during the calibration session, the 30th percentile of the positive ΔHbO_2_ values as the target level to reach 100% during the NF session. This value will be stored and used to compute the fNIRS NF score:


$$\;NF_{fNIRS}\;=\;\frac{diff\Delta HBO_{2}\;(NF\;task)}{diff\Delta HBO_{2}\;(calibration)}*100.$$

We focus specifically on the expected response during movement, an increase in the relative ΔHBO_2_ concentration during the task is expected, which is reflected by a positive ΔHbO_2_ [55].

**EEG-fNIRS-NF score.** The EEG-fNIRS combined score is based on the average of the *NF*_*EEG*_ and *NF*_*fNIRS*_ scores. It is refreshed every time a new EEG or fNIRS score is calculated:


$$NF_{EEG\text{-}fNIRS}\;=\;\frac{{NF}_{EEG}+{NF}_{fNIRS}}{2}.$$

#### 2.1.4. *Implementation.*

The signals and scores are transmitted between recording and processing applications using the Lab Streaming Layer protocol (LSL, San Diego, USA, v1.16.0, Stenner et al., 2022 [56]).

**Acquisition and real-time preprocessing**. All the real-time pre-processing steps and their implementation are presented in Fig 3. Our platform is using the OpenViBE software (Renard et al., 2010 [57], modified version 3.6.0-NIRS, accessible on the git repository of the platform) to perform the acquisition for the EEG and the real-time pre-processing steps for both EEG and fNIRS signals and real-time pre-processing. The EEG is acquired using the OpenViBE Acquisition server software which implements drivers for the Brain Products ActiCHamp amplifier (OpenViBE configuration: 25 samples sent per block of signal). The EEG data is sampled at 500 Hz with Fpz as the ground electrode. For fNIRS signal acquisition, the NIRStar software is used (v15.3, NIRx, Berlin, Germany) to send the raw signal to the OpenViBE acquisition server software through LSL (OpenViBE configuration: 1 sample sent per block of signal). The fNIRS signal is sampled at 6.25 Hz with 10 sequential steps of LED sources lighting (1: S1, 2: S2, S13; 3: S3, 4: S4, S15; 5: S5, S12; 6: S6, S9; 7: S7, S11; 8: S8, S10; 9: S14; 10: S16). Once entered into the OpenViBE Designer software, the fNIRS and EEG signals undergo several processing steps as described in the previous section. For the fNIRS preprocessing steps, we adapted to real-time processing the MNE functions (MNE-Python, Gramfort et al., 2013; MNE-NIRS, Luke et al., 2021 [58,59]) that perform the conversion to optical density (mne.preprocessing.nirs.optical_density), the conversion to hemoglobin states (mne.preprocessing.nirs.beer_lambert_law) and the short channels correction (mne_nirs.signal_enhancement.short_channel_regression). The localization of the long and short channels (i.e., x, y, z coordinates, obtained from the “probe-info.mat” file generated by NIRStar) is extracted to identify the nearest short-channel to each long-channel.

**Fig 4:**
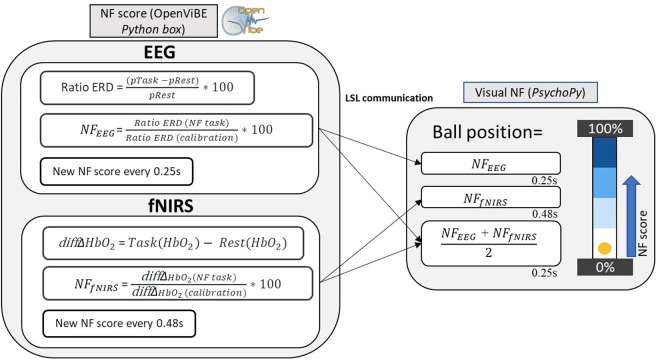
Implementation of NF score computing and visual representation. *The calibration score* ${dif\;\;\;f\Delta HbO}_{2}$ obtained during the calibration session. With the epoching, a new score is computed every 0.25 s for the EEG and every 0.48 s for the fNIRS.

**Neurofeedback score metaphor.** The implementation of the score metaphor is presented on Fig 4. We chose a visual feedback adapted from the work of Perronnet et al. (2020) [28]. The NF paradigm includes the use of a visual metaphor consisting of a vertical gauge with a yellow ball moving up and down in 1D. The ball position represents the EEG scores, fNIRS scores or the average of both EEG and fNIRS scores. The background of the 1D gauge is divided into four regions to provide the subject with reference points. The NF score calculation is carried out using a dedicated Python code (Python 3.12). Visual instructions and feedback are presented to the user using the PsychoPy toolbox (Peirce et al., 2019, version 2023.2.3 [60]). The ball position ranges from 0% to 100%. If the NF score exceeds 100%, it is capped at 100%, and if it is negative, it is set to 0%. Both the *NF*_*EEG*_ and *NF*_*fNIRS*_ score is transmitted to the Python Psychopy script through LSL and the ball position is updated accordingly. Every time a new score arrives, the ball position is updated.

**Fig 5:**
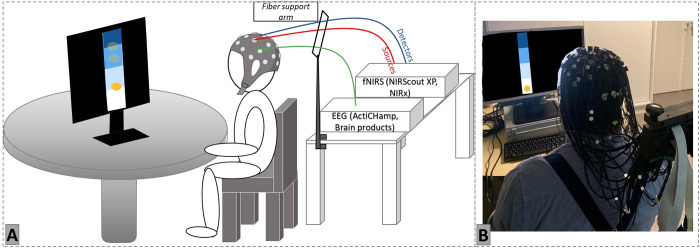
Final set-up EEG-fNIRS-NF experimental platform. **A.** Schematic representation of the installation of a participant in a dedicated dark room (to avoid light noise). The fNIRS (NIRScout XP, NIRx) and EEG (Actichamp, BrainProducts) systems are connected to the experimenter’s computer to launch acquisition and signal feedback. **B.** Picture of a pilot participant with the complete cap set-up with the cables lifted by the fiber support arm.

**Fig 6:**
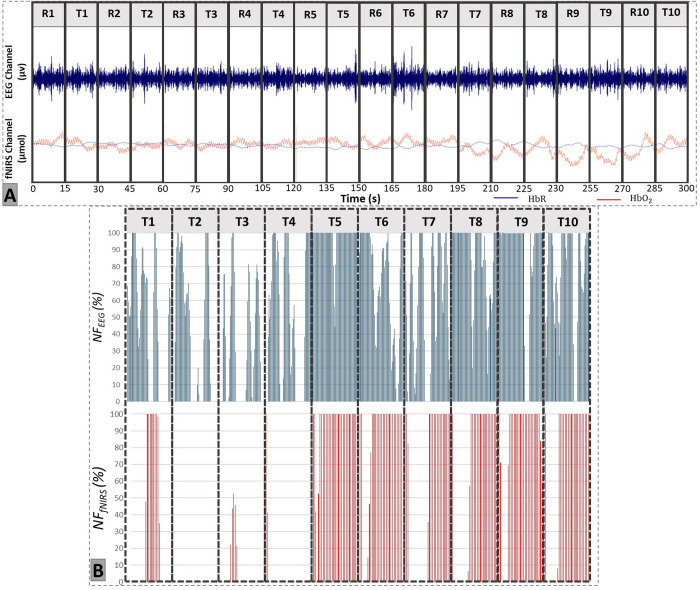
Synchronization of the several steps on the EEG-fNIRS-NF experimental platform. **A.** EEG and fNIRS raw signals acquisition. Alternance of 15 s rest (R) and 15 s NF-trial (T) periods. **B.** NF_EEG_ and NF_fNIRS_ scores calculation for all of the 10 NF-trials **(T)**.

**Final set-up.** The experimental setup is illustrated in Fig 5. The cables of the EEG and fNIRS system could be lifted using a mechanical support arm (Fiber Arm, NIRx) to reduce the weight on the head and minimize the possibility of cap movement. Technical tests showed that the platform works as expected: it simultaneously records the fNIRS and EEG signals, calculates the NF scores and updates the visual feedback accordingly. The synchronization of these several steps is presented on Fig 6.

### 2.2. Study protocol

We present now the study protocol we propose to assess the benefit of multimodal EEG-fNIRS-NF with the platform previously presented. This study was approved by the National Ethics committee of Inria (COERLE, Inria Operational Committee for the Assessment of Legal and Ethical Risk, nº 2024−27). The data acquisition started 1st of December 2024 and is running until 30th June 2025. Results of the acquisition are expected by September 2025.

#### 2.2.1. *Participants.*

Thirty healthy adults will be recruited. The inclusion criteria are i) 18-year-old or older, and ii) right-handed (score > 0.5 Edinburgh inventory, Oldfield, 1971 [61]). The non-inclusion criteria are i) to report a neurologic or psychiatric disease, ii) to report left upper limb (UL) orthopedic issues. All participants will have to read an information letter and sign an informed consent form before participating in the experiment.

#### 2.2.2. *Study design.*

**Fig 7:**
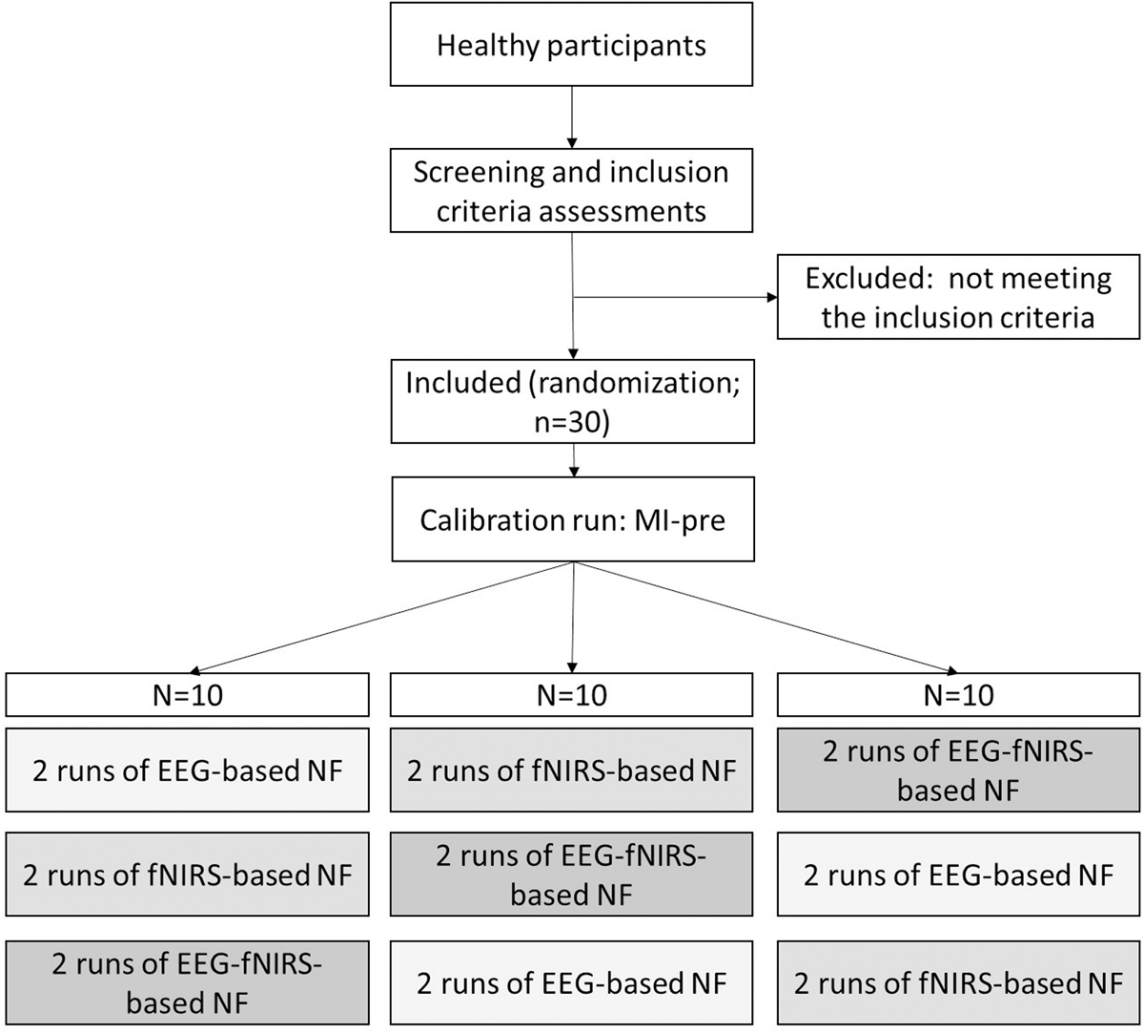
Flow chart of the study design. After checking the inclusion criteria, participants will undergo three randomized conditions of NF (EEG-based, fNIRS-based, EEG-fNIRS-based), with two runs for each condition.

This is an interventional randomized controlled study. All participants will undergo a single 90 minutes session with 45 minutes of installation and questionnaires and 45 minutes of experiment (with ~32.5 min of NF and MI task). The experiment will take place in a quiet isolated room. The participants will be equipped with our fNIRS-EEG neuroimaging system (see detailed description in section *2.1.*1. Cap-layout**) and then will perform a MI task with a visual NF in three experimental conditions repeated two times: fNIRS-based NF, EEG-based NF and EEG-fNIRS-based NF. The order of the three conditions will be randomized with control for age and gender. Participants will be blinded to the neuroimaging condition as both EEG and fNIRS systems will be set up and record brain activity during the whole session. **Fig 7** shows a flow diagram of the study design.

#### 2.2.3. *Data acquisition procedure.*

**Preparation.** Participants will first receive an oral explanation of the objectives of the study and their individual characteristics will be assessed (i.e., MI abilities, sleepiness level, hours and quality of sleep, experience with MI or NF and regular engagement in physical or artistic activities involving the upper limbs; see the “Data and Outcomes” section for more details). Next, participants will be equipped with our EEG-fNIRS system. The participant is comfortably sitting in a chair (with upholstery), in a calm room and positioned in front of a table, facing a 27-inch screen placed approximately 80 cm from their heads. The NF tasks are performed in the dark, but, between all runs, the light is turned on. Finally, to ensure participant comfort, between each run, they have the possibility to move a bit on the chair and to drink a glass of water. They will be instructed to place their feet flat on the floor and rest their hands on their thighs to minimize unintentional hand movements during the MI task. Throughout the session, participants will be asked to remain as immobile as possible, with a particular emphasis on limiting head movements. To ensure the quality of the real-time signal, we will use the calibration option in NIRStar software (version 15.3, NIRx) and adjust the sensors until most channels meet the acceptable tolerance (i.e., no more than 10% of bad channels). We will then start the fNIRS recording to visually check for the presence of the cardiac pulse in the ΔHbO_2_ signal. Afterward, the EEG channels will be filled with gel (Super Visc, Brain Vision Solutions) and the impedance of the channels will be measured using the BrainVision Recorder software (version 1.26, Brain Products GmbH). Finally, the real-time EEG signal will be visually checked to confirm the presence of eye artifacts on the prefrontal electrodes and muscle artifacts from jaw clenching.

**Fig 8:**
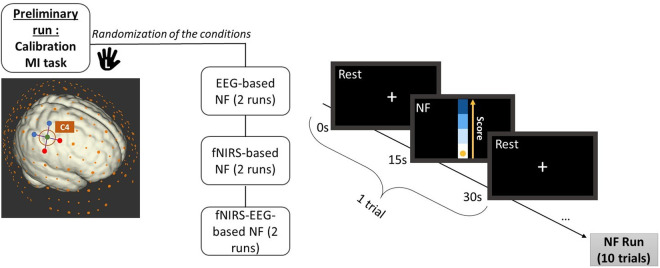
The experimental protocol will begin with a preliminary motor imagery run without NF for calibration, consisting of five alternating 15-second rest and task periods (MI task of the left upper limb). This will be followed by three conditions: two EEG-fNIRS runs, two EEG-based NF runs and two fNIRS-based runs, in randomized order across the participants. Each run will follow a block design, consisting of ten alternating 15-second rest and task periods. The NF score will be calculated based on the brain activity over the right primary motor cortex), corresponding to C4-EEG electrode and D1-S1/D2-S2 FNIRS channels in our layout.

**Instructions and task.** Participants will be instructed that, during the NF runs, they will see a ball moving along a one-dimensional vertical gauge. They will be asked to raise and maintain the ball as high as possible by imagining movements of their left hand. To encourage self-learning, only implicit instructions will be provided, allowing participants to explore and develop their own strategies [62]. These instructions will be displayed in written form on the screen at the beginning of each NF run (instructions available on the git repository). More specifically, participants will be instructed to perform both kinesthetic and visual MI of their left hand to control the ball [63]. Engaging the non-dominant hand in right-handed individuals may require more focused attention, thereby increasing cognitive load and promoting neural plasticity [64]. Kinesthetic MI involves attempting to feel the motion while visual MI involves imagining the movement as vividly as possible [65]. The experimental protocol will consist of two phases. First, participants will complete a preliminary MI run without NF (MI-pre) to calibrate the NF scores. Second, they will perform two NF runs for each of the three conditions: EEG-based, fNIRS-based, and EEG-fNIRS-based NF. A one-minute break will be given between the two runs and a five-minute break will be provided between conditions. During the five-minute break, participants will fill out a questionnaire on the experimental computer. Each of the six NF runs will follow a block design alternating between ten 15 s rest periods and ten 15 s task periods, resulting in a 5 minutes run. The calibration session will alternate between five blocks, resulting in a 2.5 minutes lasting run. During rest periods, the screen will display a white cross and participants will be asked to focus on the cross and disengage from the MI task, allowing their thoughts to drift (see Fig 8).

**Neurofeedback evaluation.** In accordance with the CRED-nf checklist [66], the subjective feeling of success and the mental strategies used to control the ball movement will be gathered during an interview after the two runs of each NF condition. Participants will have to fill out a custom-made form asking: i) the type of movement imagined during the runs; ii) the strategy used for the task (open question); iii) the level of the MI kinesthetic and visual information; iv) the level of sleepiness; v) the feeling of controlling the NF; and vi) the feeling of ability to disengage from the MI task at rest.

#### 2.2.4. *Data and outcome measures.*

All outcome measures are presented in Table 1, organized according to the timing of the experiment (before the experiment, calibration session, EEG-based NF condition, fNIRS-based NF condition and EEG-fNIRS-based NF condition). Additionally, we will collect participants’ age, gender as well as their previous experience with NF and MI, their use of the left upper limb in specific physical or artistic activities (e.g., music, sports), the quality and quantity of sleep before the experiment and their awareness level.

**Table 1:** Study outcomes assessments before the NF experiment (column “Pre-NF”), during the calibration of the MI task (column “Calib”) and for each experimental condition (columns “EEG-NF”, “fNIRS-NF” and “EEG-fNIRS-NF”).

Outcomes measures	Pre-NF	Calib	EEG-NF	fNIRS-NF	EEG-fNIRS-NF
Quantity and quality of sleep	X				
MI vividness (KVIQ)	X		X	X	X
Awarness level (Stanford scale)	X		X	X	X
VAS – feeling of controlling the gauge			X	X	X
VAS – feeling of disengagement at rest			X	X	X
Strategies used to do the NF task			X	X	X
Brain activity (EEG/fNIRS)		X	X	X	X
NF scores			X	X	X

**Motor imagery abilities.** We will assess participants’ general MI ability before starting the experiment using the short version of the Kinesthetic and Visual Imagery Questionnaire (KVIQ-10, Malouin et al., 2007 [67]). The KVIQ evaluates the vividness of MI for five simple movements. For each movement and MI modality (visual and kinesthetic), participants will be asked to: i) physically perform the movement, ii) imagine the movement and then self-rate the vividness of their MI on an analog scale from 1 (no image or kinesthetic sensation) to 5 (image as clear as during the actual movement, with similar perceived sensations). Participants will also self-report their MI vividness after each NF condition using the same visual and kinesthetic scales as those in the KVIQ-10.

**Sleepiness level.** Before starting the experiment and after each condition, participants will rate their sleepiness level using the Stanford Sleepiness Scale [68], with a range from 1 (fully awake) to 8 (sleeping). Prior to the experiment, participants will also report the sleep quality from the previous night on a Likert scale from 1 (very poor) to 5 (very good) and the number of hours they slept.

**Mental strategies for neurofeedback.** After each of the three NF conditions, participants will complete a questionnaire about the strategies they used during the task. Questions will be: “What type of movement did you imagine to move the ball?” and “Describe the strategy you used to perform the task” (e.g., ball fixation, focus, self-encouragement, visualization, etc.). Additionally, participants will rate their feelings of control over the gauge and their ability to disengage from the MI-NF task using a visual analogue scale (VAS). They will be asked: “Did you feel like you were in control of the gauge?” and “Did you feel it was easy to disengage from the task?” The VAS consists of a straight line, where 0 represents “not at all” and 10 represents “totally”.

**Neurofeedback scores.** All fNIRS-based and EEG-based NF scores (*NF*_*fNIRS*_, *NF*_*EEG*_ and *NF*_*fNIRS-EEG*_) will be stored and organized according to the condition (EEG, fNIRS, EEG-fNIRS) and the run [1,2]. Within each run, all scores will be averaged to produce a global NF score per condition and per run. Moreover, the time course of the scores will be extracted. The process for calculating these scores is outlined in the section Neurofeedback score.

**Brain activity.** Sensorimotor cortical region activation will be measured during the seven steps of the experiment (MI pre and two runs for each of the three conditions). For offline analysis, the fNIRS signal will be preprocessed in the same manner as during real-time processing (see Section fNIRS online processing), with the exception of a more detailed inspection of the channel quality, and the use of the whole signal for optical density conversion, short-channel correction and HB states. For the quality check, we will assess the scalp-coupling index and peak spectral power for each channel, condition and run [69–71]. Channels that meet the quality check criteria will be included in the offline analysis. We will examine changes in the average, peak and area under the curve of ΔHbO_2_ and ΔHbR concentration in the contralateral (right) and ipsilateral (left) sensorimotor cortical regions during the three conditions (EEG-NF, fNIRS-NF, EEG-fNIRS-NF). For offline analysis, the EEG signal will be preprocessed in the same manner as during real-time processing (see Section EEG online processing), but adding an independent component analysis to better remove extra-cortical activity such as eye blinks, Hyvarinen, 1999 [72]). The changes in sensorimotor cortex neural oscillations will be assessed by examining the magnitude and ratio of alpha- and beta-frequency power in both the ipsilateral and contralateral motor cortical regions during rest and the NF task. The event-related desynchronization (ERD, power decrease during the task) and event-related synchronization (ERS, power increase during rest) of the alpha motor mu rhythm (8–13 Hz) and beta rhythm (13–35 Hz) will be calculated for each run and averaged according to the condition (EEG, fNIRS, EEG-fNIRS) and run [1,2].

#### 2.2.5. *Statistical analysis.*

Statistical analysis will be performed using the latest version of the R software. A significance level of p < 0.05 will be used for all analyses. The primary outcome will be the difference in the brain activity between the three conditions: EEG-based NF, fNIRS-based NF and EEG-fNIRS-based NF. To check that the participant has succeeded, at least to some extent, in performing the neurofeedback task in each condition, we will study the NF scores. The exploratory outcomes will include the perception of control over the gauge and the strategies employed in relation to the NF condition as well as the link between the MI abilities and the performance at the NF-task. To assess the normality of the NF scores and brain activity data (i.e., EEG-ERD, EEG-ERS, fNIRS-HbR, fNIRS-HbO_2_), the Shapiro-Wilk test will be conducted. If the data are normally distributed, parametric repeated measures ANOVA will be used for mean comparisons. In cases where the data do not follow a normal distribution, non-parametric Friedman tests will be employed to evaluate differences among conditions. The perception of control over the gauge, as a function of the NF condition, will also be compared using the Friedman test. Lastly, as an exploratory analysis, Pearson correlation coefficient will be used to examine the relationship between the feeling of NF control, the MI abilities and the NF scores, depending on the NF condition.

#### 2.2.6. *Data management plan.*

The participant will only be identified by a unique identification number. An identification list of subjects will be kept in the investigator’s file. The investigator will ensure that the anonymity of each person participating in the study is guaranteed. Information will be collected for each participant in a standardized observation booklet filled out by the investigator. The data will be stored on secure servers using the Shanoir web platform (https://project.inria.fr/shanoir/), which operates in compliance with the GDPR (i.e., General Data Protection Regulation of the European Union). If participant’s consent is withdrawn, the data already collected will be destroyed at the participant’s request. On the results paper of the study protocol, the de-identified data will be shared, in accordance with the ethic committee of the Inria laboratory (COERLE, Inria Operational Committee for the Assessment of Legal and Ethical Risk, nº 2024−27).

## 3. Discussion

The aim of this paper was to present the experimental platform developed for EEG-fNIRS-based NF and to propose a study protocol that will be conducted to evaluate the effects of multimodal EEG and fNIRS NF for MI of the left upper limb in comparison to unimodal EEG-NF and fNIRS-NF.

**Experimental platform for multimodal NF.** The design of the NF platform was based on prior work with EEG-NF [27,73,74,75] and existing literature on fNIRS-NF [22,23,52]. This platform relies on a modular architecture and offers the possibility of distributing the system on different processing units, providing either EEG only, fNIRS only or combined EEG-fNIRS scores. This paper proposes a methodology that addresses the technical challenge of integrating EEG and fNIRS systems for real-time brain activity analysis. The choice to combine both EEG and fNIRS information into a single neurofeedback (NF) stream was motivated by previous research showing that participants find it easier to control a single mixed feedback signal, rather than multiple separate signals [27]. Additionally, because the fNIRS hemodynamic response is delayed by a few s, presenting the signals separately could lead to reduced performance, as participants may focus on the faster-changing EEG feedback.

Positioning the fNIRS channels and EEG electrodes was crucial for our cap design as we aimed to record the EEG and fNIRS responses originating from the same cerebral location which implies an overlapping position for the EEG electrode and the fNIRS channels. For the rest of the cap, to optimize the set-up and its mobility, we privileged a maximal coverage of the pre-motor, sensorimotor and primary motor brain area for both EEG and fNIRS. The pre-processing steps and NF score extraction were designed in line with the literature [22,23,28,52,73,74,76]. To take into account extracerebral activity confound, we enhanced our pipeline through/with a real-time short channel correction. But one limitation here is to use only the ΔHbO_2_ signal as NF-fNIRS parameters without including ΔHbR in the score calculation [52]. This choice was made based on previous works where ΔHbO2 was used successfully to calculate changes in hemodynamic activity in the right-M1 in healthy young adults [77,78] and individuals with pathologies [79,80]. Moreover, according to the Hemodynamic Response Function (HRF), evoked changes in cerebral oxygenation, i.e., increased HBO_2_, reflect increased brain activity [48]. The HbR signal will be exploited together with the HbO2 signal for the offline analysis.

A technical test confirmed that our platform operates reliably (i.e., easy to use, without lags, recording all data points), and in particular that EEG and fNIRS signals can be acquired simultaneously and processed in real time to provide unimodal or multimodal NF scores.

**Study protocol to evaluate multimodal neurofeedback.** Our protocol will be the first trial to evaluate the impact of multimodal NF with EEG and fNIRS for upper-limb motor imagery. In this study, we will implement integrated NF requiring participants to regulate both EEG and fNIRS simultaneously to reach the NF target. This approach assumes that both signals can be combined since neurovascular studies demonstrate a correlation between electrophysiological (EEG) and hemodynamic (fNIRS) activity [81]. What makes multimodality particularly interesting is that these methods provide complementary information regarding brain functional activity during upper limb movements [80,82,83]. Both EEG-based NF (EEG-NF) and fNIRS-based NF (fNIRS-NF) have been explored separately in both healthy and clinical contexts (for reviews on EEG, see [84–86] Viviani & Vallesi, 2021; Patil et al., 2023; Cheng et al., 2024; for reviews on fNIRS, see [22,52,87]. As in Godet et al. (2023) [49], we followed the CRED-checklist [66] and NF recommendations for the design of the protocol and its evaluation with for instance the questions asked to the participants at the end of each condition about the strategies and feeling of control. To optimize the system, the use of MRI or neuro-navigation system would be beneficial to locate the brain regions of interest. With our current system, will only use the 10−10 international system reference, which could lead to lower spatial accuracy. Since all sensors are set upon the same layout, it will not be possible to cover exactly the same brain areas with the EEG and fNIRS, but for NF robustness, we designed the cap to cover exactly the same brain area for the NF target. This was achieved by putting the EEG C4 channel in the middle of 2 sources and 2 detectors. A compromise needs to be made when implementing EEG and fNIRS for real-time applications, due to the difference in temporal resolution between the two modalities. Indeed, the frequency of the fNIRS score calculation is lower than that of the EEG, in line with the temporal dynamics of fNIRS and EEG (neurovascular response or electrical response respectively). However, this was done in order to get the best out of both systems, without refreshing the EEG too often, so that the NF is not mainly guided by it, and with a slightly higher refreshing frequency of the fNIRS, to take advantage of its resolution.

Finally, the design of the NF protocols plays a critical role in shaping the underlying neural mechanisms that support learning and plasticity. NF is thought to promote plastic changes through repeated, reinforced activation of specific brain circuits, consistent with principles of operant conditioning and Hebbian learning [3,88]. In our study protocol, we chose a block design in order to facilitate stronger and more consistent activation of targeted neural networks over time, promoting experience-dependent synaptic modification and functional reorganization [2]. This block design, coupled with real-time feedback and repeated training, creates the conditions for neural adaptation and learning, even if the exact causal pathways remain an active area of investigation [3].

The designed protocol aims to achieve an increased brain activity in the right primary motor cortex, the participants being instructed to mentally simulate left hand movement. Nevertheless, as the strategy used will only be subjectively reported, we will not be able to really track if the strategy might change from block to block and the subjective report will not necessarily refer to the whole condition. The reporting strategies will nevertheless give us a general idea of the most successful strategy used, and via the quality of the MI (using KVIQ scale, [67]), we will be able to see if participants feel they have mastered mental movement simulation.

**Perspectives for clinical applications.** With the potential to enhance neuroplasticity, such EEG-fNIRS-NF system could have significant applications in clinical settings, particularly in motor and brain rehabilitation. A deeper understanding of its benefits could be achieved by examining neurovascular coupling (NVC). Indeed, altered NVC is common after stroke, making its assessment highly relevant for interventions aimed at stimulating neuroplasticity and thus motor recovery. NVC describes the interaction between electrical neural activity (excitatory or inhibitory), cerebral blood flow (hemodynamic responses), and the brain’s metabolic energy consumption [89]. This coupling leads to hemodynamic changes in brain regions that reflect electrical activity, characterized by a localized increase in blood flow [90]. As such, NVC is essential for understanding brain function and dysfunction. In this context, combining EEG and fNIRS has been shown to be particularly effective for elucidating NVC processes in humans [82]. Since both EEG and fNIRS can be recorded simultaneously without interfering with one another (i.e., not degrading one signal or the other, as in EEG-fMRI, Ihalainen et al., 2015 [91]), they offer complementary insights into electrical and hemodynamic brain activities at improved resolution [41,92]. Such a platform could be easily implemented in clinical practice. Recent decades, fNIRS and EEG methods have been used in several stroke studies [93]. This relies on providing an accessible, affordable system, robust, easy to install and use – what we had in mind when developing the software. Our future findings could come to reinforce their interest, especially when associated with functional evaluation. A long-term objective of this study would be to explore the impact of this multimodal system on post-stroke rehabilitation, building on previous findings that demonstrate the motor recovery benefits of neurofeedback methods [9].

## 4. Conclusion

This paper presented an experimental platform developed for multimodal neurofeedback with EEG and fNIRS. It also presents a study protocol designed to evaluate the benefits of EEG-fNIRS combination for NF in the context of upper-limb MI in healthy population. These modalities have shown great potential in clinical applications, and their combination for NF is promising. To conclude, the benefit of this study could be a better efficiency of the NF protocol without changing the length of the programs. Indeed, the association of the two neuroimaging modalities could allow for a better neuroplasticity leading to higher brain-related global activity increasing the response to the NF with several sources of brain activity. The results of such a study will help to better understand the neurophysiological modifications associated with NF training and, in the long-term, to the post-stroke recovery related to NF training protocols.
